# Refractory tonic-myoclonic status epilepticus with catamenial recurrence in epilepsy with myoclonic atonic seizures: A case report

**DOI:** 10.1016/j.heliyon.2024.e24747

**Published:** 2024-01-14

**Authors:** Jacopo Proietti, Elena Fiorini, Gaetano Cantalupo, Elena Fontana, Tommaso Lo Barco, Cecilia Bonin, Bernardo Dalla Bernardina, Francesca Darra

**Affiliations:** a UOC Neuropsichiatria Infantile, Dipartimento Materno-Infantile, Azienda Ospedaliero-Universitaria Integrata, Verona, Italy - Full member of ERN EpiCARE; b Innovation biomedicine Section, Department of Engineering for Innovation Medicine, University of Verona, Verona, Italy; c Center for Research on Epilepsies in Pediatric age (CREP), Verona, Italy; d U.O.C. Ostetricia e Ginecologia B, Dipartimento di Ostetricia e Ginecologia, Azienda Ospedaliera Universitaria Integrata, Verona, Italy

**Keywords:** Epilepsy with myoclonic-atonic seizures, Doose syndrome, Status epilepticus, Post-puberal age, Catamenial, Progestin

## Abstract

In epilepsy with myoclonic-atonic seizures (EMA), status epilepticus (SE) may occur during the onset phase, uncommonly in post-puberal patients. We report a post-puberal patient with EMA who presented SE with insidious onset and catamenial recurrence.

She had a stormy epilepsy onset at 4 years, with tonic seizures, atypical absences, and myoclonic-atonic seizures, in the absence of SE. After the onset phase, sporadic nocturnal tonic seizures persisted and a mild intellectual disability appeared. At the age of 7, after gonadotropin-releasing hormone analog administration due to central precocious puberty, she presented with SE characterized by recurrent atypical absences, tonic seizures, and awareness impairment, which was successfully treated in 4 days. At 11 years, one week before menstruation, the patient presented with analogous SE that lasted 8 days.

One week before the subsequent menstruation, she presented again with SE, initially characterized by atypical absences alternating with phases of awareness and motor impairment related to fast low-voltage EEG activity in the central regions; later, tonic and myoclonic seizures occurring even in the awake state increased, and the “atonic-akinetic status” related to fast EEG activity worsened. After conventional antiepileptic drugs had failed to control the seizures, a progestin was added, with subsequent gradual complete recovery.

## 1 Introduction

Epilepsy with myoclonic-atonic seizures (EMA) is a syndrome characterized by the presence of myoclonic-atonic seizures in an otherwise normal child who may have a history of febrile and/or afebrile seizures and a family history of seizures [1]. At the onset of the disease, between 6 months and 6 years of age, there is a variable association of different seizure types: generalized tonic-clonic, myoclonic, and myoclonic-atonic seizures are hallmarks of the syndrome, while clonic seizures, atypical absences, and tonic seizures are also often observed [2,3]. Non-convulsive status epilepticus can occur. It is characterized by stupor, apathy, drooling, and speech disorder associated with background EEG slowing and with subcontinous spikes and slow waves associated with erratic myoclonias and interposed massive myoclonias and atonic phenomena. This status, demonstrating an epileptic encephalopathy, mainly occurs during the onset phase, while it is rare post puberty [4,5]. Here we report the case of a 12-year-old patient affected by EMA who presented status epilepticus with peculiar electroclinical features only after puberty, with catamenial recurrence.

## 2 Case report

The patient had epilepsy onset at the age of 4 years when she was otherwise healthy. At that time, she had isolated tonic seizures 3 times in 40 days. These persisted with a plurimonthly to weekly frequency in the following months, when atypical absences and myoclonic-atonic seizures occurring up to several times a day became evident, in the absence of status epilepticus (SE). A developmental assessment performed with Griffith's Scale at the age of 4 years 3 months showed a General Quotient of Development (GQ) = 96. Cerebral MRI was unremarkable. *SLC2A1* and *SCN1A* genetic tests were negative. A diagnosis of EMA was made and antiepileptic therapy was started, with valproate (from the age of 4 years 3 months), ethosuximide (from 4 years 5 months), and clonazepam (from 4 years 10 months). Periodic neuropsychological assessment was performed in the following years; at age 7 a mild intellectual disability was detected. By age 6 her seizures while awake remitted, while myoclonic seizures and sporadic tonic seizures during sleep persisted. At 6 years 9 months, because of persistent hyperammonemia, valproate was stopped and changed to levetiracetam.

At 7 years 2 months the girl was diagnosed with central precocious puberty, and treatment with a gonadotropin-releasing hormone analog was planned. One week after the first drug administration the patient presented a SE characterized by recurrent atypical absence seizures and tonic seizures, with progressive impairment of consciousness. These were successfully treated in 4 days with hydrocortisone and a shift from clonazepam to clobazam. The treatment with gonadotropin-releasing hormone analog was suspended, as it was suspected to be related to the SE. The girl had her menarche at the age of 8 years 6 months.

Nocturnal tonic seizures persisted, recurring 3–4 times per month to several times a week, more frequently in the peri-menstrual period. The interictal EEG was stable over time and characterized by rhythmic theta activity in the centro-parietal regions in absence of epileptiform abnormalities in the awake state, with rare high-amplitude generalized spike-wave or polyspike–slow wave complexes during sleep; these were sometimes associated with myoclonic jerks. At age 10 the clobazam dose was slowly reduced.

At 11 years 2 months, one week before menstruation, the patient presented a second SE characterized by atypical absences, recurrent episodes of awareness impairment with fluctuating fast low-voltage EEG activity in the central regions, and a concomitant increase in the frequency of myoclonic and tonic seizures during sleep. These remitted in 8 days with hydrocortisone and clobazam, both later withdrawn at 3 weeks.

**Fig. 1:**
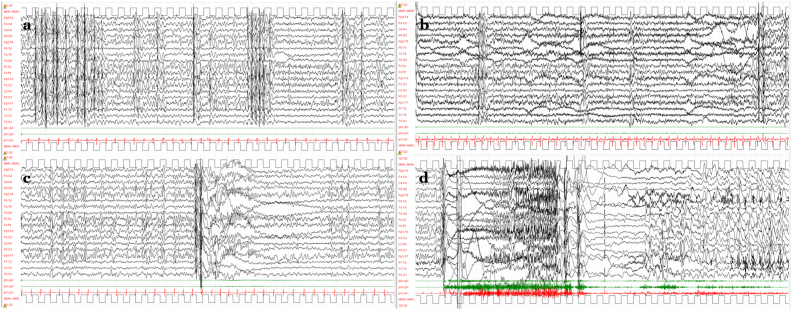
EEG findings in the earliest days after admission. A) In the awake state theta-delta monomorphic background activity predominates in the centro-parietal areas, with superimposed generalized spike/polyspike-wave discharges (the clinical correlate of atonic phenomena involving face and neck muscles is not evident on the polygraphic channels here exploring deltoids). B) Low-amplitude subcontinuous fast activity or polyspike discharge predominant in the motor areas appears in some phases, clinically related to awareness and motor impairment fluctuating from motor and speech slowing to a hypomotor state with obtundation, drooling, and aphasia. C, D) During sleep, myoclonic seizures characterized by a high-voltage polyspike complex (associated with a sudden and brief muscle contraction, recorded on the polygraphic channels) followed by a slow wave (C), and tonic seizures—low voltage recruiting polyspike discharge with concomitant diffuse tonic posturing ending with brief clonic phase (D)—were recorded. [Voltage gain 100 μV/cm; time resolution 1,5 cm/s; high-pass filter 1.6 Hz, low-pass filter at Hz.].

The following month, one week before the subsequent menstruation, the patient presented again with SE, initially analogous to the previous one. EEG recordings showed a global slowing of background activity with theta-delta monomorphic activity predominant in the centro-parietal areas, with superimposed frequent generalized spike/polyspike–wave discharges associated with atonic phenomena involving mimic muscles and sometimes subtle head drops or blinking (Fig. 1A). In some phases the EEG was further modified by the appearance of a low-amplitude subcontinuous fast activity or polyspike discharge, predominant in the motor areas and alternating with the generalized spike/polyspike-waves. Clinically the picture was characterized by an awareness and motor impairment fluctuating from motor and speech slowing to a hypomotor state with obtundation, drooling, and aphasia (Fig. 1B). Myoclonic seizures and tonic seizures were recorded during sleep (Fig. 1C and D). In combination with the in-place therapy (ethosuximide and levetiracetam), acetazolamide was started.

**Fig. 2:**
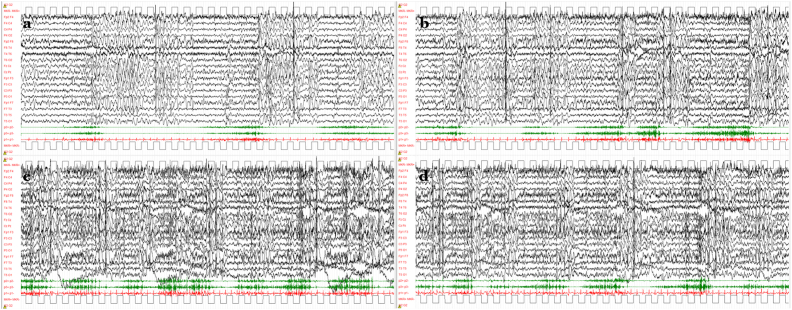
Evolution of the EEG fast-activity sequences observed in the awake state from day 4 after admission. A, B) Low-amplitude fast activity or polyspike discharge in the motor areas associated with bilateral increase in muscle tone, regressing in combination with the appearance of generalized spike/polyspike-waves. C, D) These tonic episodes could be mixed with myoclonic and myoclonic-atonic features, as shown by the polygraphic recordings of deltoid muscles. [Voltage gain 100 μV/cm; time resolution 1,5 cm/s; high-pass filter 1.6 Hz, low-pass filter at Hz.].

Four days later, the EEG fast activity sequences evolved into more obvious seizures associated with bilaterally increased muscle contraction with concomitant myoclonic and myoclonic-atonic phenomena (Fig. 2A–D); these initially receded with a single dose of clonazepam. Contemporary, tonic seizures occurring even in the awake state increased in frequency. Acetazolamide was suspended due to inefficiency, and oral clonazepam and intravenous hydrocortisone were added to the background therapy.

**Fig. 3:**
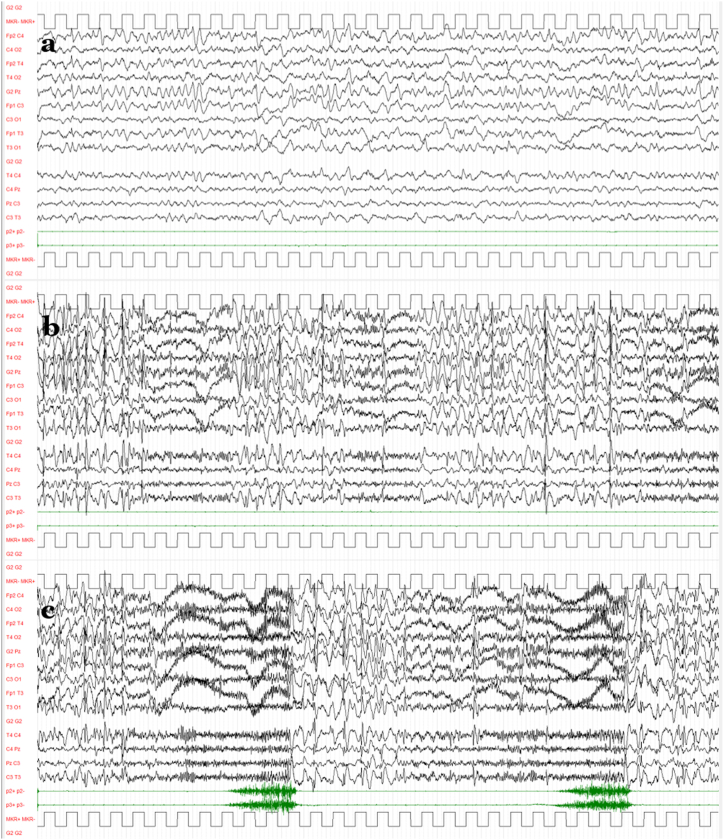
The three distinct and periodically alternating EEG patterns observed while the patient was in the intensive care unit. A) Sinusoidal theta activity in the central regions. B) Diffuse spike-waves intermixed with fast tonic discharge. C) Tonic seizures in recurring clusters with variable intensity and duration. [Voltage gain 100 μV/cm; time resolution 1,5 cm/s; high-pass filter 1.6 Hz, low-pass filter at Hz.].

In the following days the patient worsened further: sub-continuous fast activity or polyspike activity became more continuous and the “atonic-akinetic status” worsened (becoming nonresponsive to benzodiazepine therapy), while tonic seizures further increased in frequency, intensity, and duration and tended to arise in clusters. Rufinamide and phenobarbital were introduced in therapy without significant benefit. The girl became dysphagic and unable to feed herself and to take oral medicine. Twelve days after hospital admission she was transferred to the pediatric intensive care unit, where midazolam continuous infusion was given for 7 days (highest dosage 6 mcg/kg/h). Intravenous loading doses followed by maintenance were set for lacosamide and phenytoin (from 14 to 16 days after admission, respectively). Afterwards, topiramate was added to the complex polytherapy regimen. A ketogenic diet was configured, but a state of ketosis was only reached 4 weeks later. The patient's condition remained stable in its severity, and the EEG monitoring showed persistent periodic alternation of three distinct patterns: sinusoidal theta activity in the central regions (Fig. 3A), diffuse spike-waves intermixed with fast tonic discharge (Fig. 3B), and tonic seizures in recurring clusters that varied in intensity and duration, with seizure-free intervals rarely exceeding 1 h (Fig. 3C).

After 19 days from the onset of SE, on the suspicion that a catamenial component could be playing a role in its genesis and maintenance, adjunctive progestin therapy with desogestrel at the dose of 75 mcg daily was started.

Later the patient showed gradual electroclinical improvement, which was particularly evident from day 24 after onset. The phases characterized by low-amplitude fast activity predominant in the motor areas became increasingly rare and shorter. The number and intensity of tonic seizures progressively declined. The last seizure in the awake state occurred one month after the onset of the SE.

**Fig. 4:**
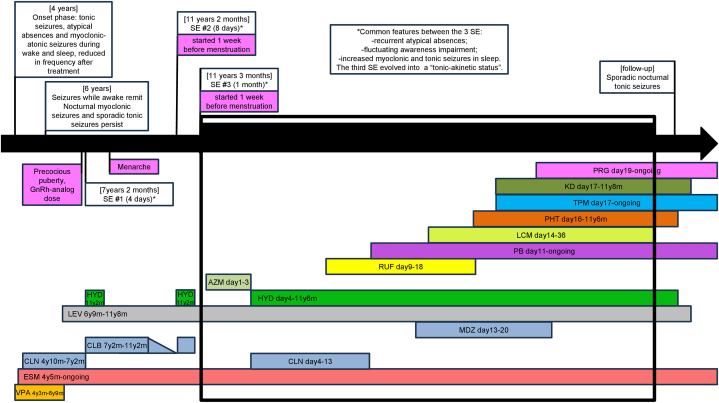
Graphical summary of the clinical evolution over time. The black arrow represents time; the age of the patient is specified in square brackets in the white boxes. Drugs abbreviations: VPA valproate, ESM ethosuximide, CLN clonazepam, CLB clobazam, MDZ midazolam, LEV levetiracetam, HYD hydrocortisone, AZM, RUF rufinamide, PB phenobarbital, LCM lacosamide, PHT phenytoin, TPM topiramate, KG ketogenic diet, PRG progestin therapy. Start and end of each ASM is specified: years and month of age are reported as nYnM, days refer to the third episode of status epilepticus.

The global clinical picture recovered completely. In the 22-month follow-up period, we observed the persistence of tonic seizures during sleep. Their semiology was characterized by a sudden jerk immediately followed by diffuse tonic posturing, predominantly of the arms and neck. They were often associated with a vibratory component and a final clonic phase and were of short duration (usually less than 5 seconds, occasionally lasting up to 20 seconds). Hydrocortisone, phenytoin, and levetiracetam were progressively suspended. The ketogenic diet was also discontinued, because of poor compliance. The patient continued treatment with phenobarbital, ethosuximide, and topiramate. The progestin therapy with desogestrel was maintained for one year and was subsequently replaced by estroprogestin treatment with ethinylestradiol and gestodene. The seizure frequency in the follow-up period ranged between 2 and 7 events per month, with the above-mentioned features, except for one month of seizure freedom. The EEG, recorded during regular outpatient visits, recovered the appearance displayed before the recurrent SE. The clinical course described is summarized in Fig. 4.

Epilepsy multigene panel testing was recently completed, and the results were negative. A molecular karyotype analysis was also performed and revealed a de novo 185-kb duplication in 7q31.31. This microduplication, with the proximal breakpoint between 119861477bp (normal) and 119892416bp (duplicated) and the distal breakpoint between 120077873bp (duplicated) and 120118805bp (normal), partially involves the *KCND2* gene (location 120273668–120750331 bp).

## 3 Discussion

This SE exhibited elements of interest in many of its features: (1) the unusual post-puberal age of onset; (2) the insidious onset, which was blurred and hard to recognize clinically, with an uncommon and difficult-to-detect EEG correlate; (3) the whole peculiar electroclinical semiology, differing from those previously reported in EMA; and (4) the strict correlation with the menstrual cycle and the response to the progestin agent after the failure of several conventional antiepileptics. The occurrence of tonic seizures is also an unusual feature in the context of EMA.

Our patient did not experience SE during the stormy onset phase of the disease, as instead frequently happens in patients with EMA [6]. Her first SE was triggered by gonadotropin-releasing hormone administered as treatment for precocious puberty, and relapses occurred 3 years later after clobazam withdrawal, with clear catamenial recurrence. At SE presentation, the interpretation of the phases characterized clinically by awareness and motor impairment was challenging, because the EEG correlate of low-amplitude subcontinuous fast activity was not obvious. The evolution, over the following days, to EEG polyspike discharges associated with obtundation and aphasia and later also with bilaterally increased muscle tone with concomitant myoclonic phenomena confirmed the epileptic nature of these phases. As a whole, the complex electroclinical semiology and evolution were much different from the non-convulsive status epilepticus described by other authors during the onset phase, which is typically characterized by background EEG slowing with subcontinous spikes and slow waves [2,5]. The interictal EEG recorded in the outpatient department, both prior to the SE and in the year that followed, revealed the absence of epileptiform abnormalities in the awake state, and only rare high-amplitude generalized spike-wave or polyspike–slow-wave complexes during sleep were detected. Thus, a transition to Lennox-Gastaut syndrome during the course of the disease was excluded.

The genetic finding of a partial duplication of *KCND2* is of dubious interpretation, since its effect on the protein is not entirely clear. The *KCND2* gene encodes the potassium channel Kv4.2. Potassium channel gating dysfunctions are associated with developmental delay and increased epileptic seizure susceptibility [7]. Traditionally, potassium channel loss-of-function has been associated with hyperexcitability disorders, but gain-of-function variants have also been described in association with epilepsy [8,9].

It is known that in many women with epilepsy the seizures are clustered around specific points in the menstrual cycle, most often around the perimenstrual or periovulatory period [10]. The pathophysiology of catamenial epilepsy remains unclear, but it is common knowledge that estrogens have proconvulsant properties while progesterone has anticonvulsant effects; a particularly strong correlation is observed between seizure susceptibility and the estrogen-to-progesterone ratio [[11], [12], [13], [14]]. Moreover, it must be considered that some ASM can exacerbate catamenial seizures; among them, phenobarbital, phenytoin, and carbamazepine are potent inducers of liver cytochrome P450 isoforms [15,16] and thus reduce the effect of endogenous neurosteroids and of progestin drugs by enhancing their metabolic processing. Comparing the seizure diary and the menstrual calendar of our patient, it is notable that in the only month during the follow-up in which amenorrhea (and thus perhaps hypothalamic–pituitary–gonadal axis inhibition) was obtained, the girl was seizure free. Our experience with this case suggests that adjunctive treatment with progesterone or estrogen antagonists may prove useful in appropriate patients.

## Parental consent

We have obtained consent from the parents for the publication of this case.

## Data availability statement

Data regarding this publication are included in the article and in figures referenced in the article. Any additional information are available from the corresponding author.

## CRediT authorship contribution statement

**Jacopo Proietti:** Conceptualization, Data curation, Formal analysis, Writing – original draft, Writing – review & editing. **Elena Fiorini:** Data curation, Investigation, Writing – review & editing. **Gaetano Cantalupo:** Data curation, Investigation, Writing – review & editing. **Elena Fontana:** Data curation, Investigation, Writing – review & editing. **Tommaso Lo Barco:** Data curation, Investigation, Writing – review & editing. **Cecilia Bonin:** Data curation, Investigation, Writing – review & editing. **Bernardo Dalla Bernardina:** Data curation, Investigation, Writing – review & editing. **Francesca Darra:** Data curation, Investigation, Supervision, Writing – review & editing.

## Declaration of competing interest

The authors declare the following financial interests/personal relationships which may be considered as potential competing interests: Gaetano Cantalupo reports a relationship with LICE (Italian League Against Epilepsy) that includes: board membership. Gaetano Cantalupo reports a relationship with SINPIA (Italian Child Neuropsychiatry Society) ’neurology section’ that includes: board membership. Francesca Darra reports a relationship with LICE (Italian League Against Epilepsy) Triveneto regional chapter that includes: board membership. All other authors declare that they have no known competing financial interests or personal relationships that could have appeared to influence the work reported in this paper.
